# The Voice and the Circulation

**Published:** 1899-11

**Authors:** 


### The Voice and the Circulation.

Skene contributes a very suggestive article on "The Voice in Diagnosis" to the *N. Y. Med. Journal* of June 10th. Among many other points he refers to the voice of mitral insufficiency as being husky, irregularly tremulous and not well sustained. This husky quality of heart disease is due to hyperemia of the vocal cords, and is the same in character as the edematous voice of nephritis, and very like the rough, harsh voice of the habitual drinker. In aortic insufficiency, on the other hand, there is anemia of the larynx, and the voice becomes high-pitched and soft in quality, as well as ill-sustained. The dehydrated blood of Asiatic cholera and cholera morbus represents itself by a high-pitched, thin voice, reduced finally perhaps to a whisper. "The weak, soft voice in acute anemia from hemorrhage is distinguished from that of dehydration by its being low-pitched, owing to relaxation of the vocal cords." Coming on suddenly or quickly, it is a most valuable sign of concealed hemorrhage, though similar voice signs are noted from fainting or sudden shock.—*Denver Med. Times.*

